# Leucine‐rich alpha‐2‐glycoprotein 1 affects bone destruction via IL‐6 in mouse periodontitis model

**DOI:** 10.1111/odi.14952

**Published:** 2024-04-24

**Authors:** Kazuhisa Ouhara, Tasuku Takemura, Yuri Taniguchi, Ryousuke Fujimori, Tetsuya Tamura, Yuki Akane, Shinji Matsuda, Yuta Hamamoto, Tomoaki Shintani, Mikihito Kajiya, Syuichi Munenaga, Tomoyuki Iwata, Tsuyoshi Fujita, Noriyoshi Mizuno

**Affiliations:** ^1^ Department of Periodontal Medicine, Graduate School of Biomedical and Health Sciences Hiroshima University Hiroshima Japan; ^2^ Department of Innovation and Precision Dentistry Hiroshima University Hospital Hiroshima Japan

**Keywords:** bone resorption, IL‐6, leucine‐rich alpha‐2‐glycoprotein 1, *Porphyromonas gingivalis*

## Abstract

**Objective:**

To investigate the production of leucine‐rich α‐2‐glycoprotein‐1 (LRG1) in periodontitis patients and its effectiveness as a new diagnostic marker for periodontitis.

**Subjects and Methods:**

In vitro experiments were conducted to analyze LRG1 mRNA expression in human gingival epithelial cells and fibroblasts via quantitative real‐time PCR. In vivo experiments were conducted to analyze LRG1 localization in periodontitis patients. The correlation between the serum LRG1 levels and alveolar bone resorption in the mouse periodontitis model was also investigated.

**Results:**

A positive correlation existed between the periodontal inflamed surface area and serum LRG1 levels (Spearman's rank correlation coefficient: 0.60). LRG1 mRNA expression in human gingival epithelial cells and fibroblasts was upregulated by *Porphyromonas gingivalis* stimulation or tumor necrosis factor‐α stimulation. Interleukin‐6 in human gingival epithelial cells and fibroblasts induced the production of LRG1 and transforming growth factor‐β. LRG1 levels in the periodontal tissue and serum in the periodontitis model were higher than those in control mice. LRG1 local administration resulted in alveolar bone resorption, whereas the administration of interleukin‐6R antibody inhibited bone resorption.

**Conclusions:**

LRG1 levels in serum and periodontal tissue are upregulated in periodontitis and are implicated in periodontal tissue destruction through interleukin‐6 production.

## 1 INTRODUCTION

The interaction between periodontopathogenic bacteria and the host immune response results in the development of periodontitis (Hajishengallis, 2015; Socransky & Haffajee, 1994). Periodontopathogenic bacteria classified as red complexes, such as *Porphyromonas gingivalis*, and their products trigger immune responses (Nakayama & Ohara, 2017), which results in the local and systemic production of physiologically active substances, such as inflammatory cytokines (Martinez‐Garcia & Hernandez‐Lemus, 2021; Pan et al., 2019). Periodontal diseases result in the destruction of local periodontal tissue and exacerbation of systemic diseases, such as diabetes mellitus, non‐alcoholic steatohepatitis, rheumatoid arthritis (RA), in addition to premature birth, low‐weight birth, Alzheimer's disease, and Buerger disease (Bourgeois et al., 2019; Laugisch et al., 2018; Paraskevas, 2016; Yoneda et al., 2012). These diseases are detected via the evaluation of serum specimens. Thus, novel biomarkers associated with these diseases must be identified for the evaluation of periodontitis to facilitate effective medical‐dental collaboration in the future.

Leucine‐rich alpha‐2 glycoprotein (LRG) is a 50 kDa glycoprotein with a leucine‐rich motif. Leucine‐rich α‐2‐glycoprotein‐1 (LRG1) is produced during hematopoiesis, particularly during the differentiation of the neutrophilic granulocyte lineage (Wang et al., 2013). LRG1 binds to endoglin, an accessary receptor, and activates transforming growth factor‐β (TGF‐β) signaling in the endothelial cells via the TGFBR2‐ALK1‐Smad1/5/8 pathway, thereby promoting cell proliferation, differentiation, and neovascularization (Hong et al., 2019). Elevated serum LRG1 levels have been observed in patients with RA (Fujimoto et al., 2015). Similarly, LRG has also been detected in the serum and lesions of patients with inflammatory diseases, such as inflammatory bowel disease, ulcerative colitis, asthma, myocardial infarction, and heart failure (Camilli et al., 2022). Furthermore, elevated LRG1 expression has shown correlations with disease activity (Camilli et al., 2022).

Periodontitis is mainly caused by inflammatory immune responses. Pro‐inflammatory cytokines, such as interleukin (IL)‐6, IL‐1β, and tumor necrosis factor (TNF)‐α, have been identified as the key molecules leading to the development of periodontitis (Pan et al., 2019). The production of LRG1 has been detected in the liver and inflammatory lesion sites (Chong et al., 2018). The expression of LRG1 is stimulated by IL‐6 or tumor necrosis factor‐α (TNF‐α) independently (Camilli et al., 2022). IL‐6 acts synergistically with TNF‐α to induce LRG expression. The secretion of glycoproteins, such as LRG1, is greatly enhanced in the presence of multiple pro‐inflammatory cytokines (Naka & Fujimoto, 2018; Shirai et al., 2009). IL‐6 and TNF‐α are cytokines that are upregulated in patients with periodontal diseases (Gokul et al., 2012; Keles et al., 2014; Turer et al., 2017). Furthermore, LRG1 expression in the gingival crevicular fluid (GCF) and serum is increased in patients with severe periodontitis. Periodontal therapy has been shown to be effective in reducing the LRG1 levels in the GCF and serum of these patients (Keles Yucel & Balli, 2021).

The periodontal inflamed surface area (PISA) is a numerical value that is used to express the state of periodontitis in the oral cavity based on bleeding on probing and periodontal pockets. It is an effective marker for evaluating the relationship with systemic diseases. Previous studies have reported that PISA is a more effective marker for evaluating the associations with various markers of periodontitis than those used previously (Onabanjo et al., 2023).

The correlation between LRG1 production in the serum and the degree of periodontal inflammation was evaluated using PISA, and its effectiveness as a periodontal disease test marker was demonstrated in this study. The effect of LRG1 on the progression of periodontal disease was also determined in a mouse model.

## 2 MATERIALS AND METHODS

### 2.1 Patients

Fifty‐four patients (50 women and 4 men; mean age: 54.04 ± 15.98 years) aged ≥20 years with at least 10 teeth in the oral cavity, including the third molars (mean teeth number: 23.62 ± 4.84), who had visited the Hiroshima University Hospital between 2019 and 2022 were included in this study. Participants who had received periodontal treatment or antibiotics in the preceding 3 months; patients with uncontrolled systemic diseases, such as diabetes mellitus, HIV, liver disease, hypertension, hyperlipidemia; and patients who had received radiation therapy, chemotherapy for cancer treatment, or immunosuppressant drugs were excluded from the study. The experimental protocol was approved by the Ethics Committee of Hiroshima University (approval No. E2022‐0281). All participants provided informed consent prior to the commencement of the study.

The periodontal examination was performed by an experienced dental clinician using a periodontal probe (Hu‐Friedy Mfg. Co., LLC, Chicago, IL, USA). The gingival margin, probing depth (PD, mean: 3.31 ± 0.84 mm), and bleeding on probing (BOP, mean: 34.4 ± 22.84%) in six sites per tooth were recorded. The stage (stage I–IV) and grade (A–D) of periodontitis were determined according to the current classification of periodontal diseases by the 2017 World Workshop on the Classification of Periodontal and Peri‐Implant Diseases and Conditions (Chapple et al., 2018). PISA was calculated for each patient using the BOP and PD measurements at each periodontal site to quantify the amount of inflamed periodontal tissue, as described in a previous study (Nesse et al., 2008). Following oral examination, GCF was collected from the buccal aspect with PD greater than 4 mm, showing radiographical bone loss and BOP in each individual using filter paper strips (Periopaper, ProFlow Incorporated, CT, USA). Before GCF sampling, the site was isolated with cotton rolls, and the supragingival plaque was gently removed with a sterile curette and air‐dried. Then, the paper strips were carefully placed into the gingival crevice for 10 s. The absorbed GCF volume of Periopaper was measured with a calibrated electronic device, Periotron 8000 (OraFlow Inc., NY, USA). The strips were immediately transferred into an individual sterile polypropylene tube, frozen, and stored at −80°C until the analysis. GCF was eluted with 100 μL of PBS per micron and used in the experiment.

### 2.2 Collection of serum and gingival tissue from the patients

Peripheral venous blood samples (10 mL) were collected from all participants and stored in a plain blood collection tube. The blood samples were centrifuged at 2000 rpm for 20 min to obtain the serum and stored at −80°C until use. All experimental procedures performed in this study were approved by the Ethics Committee of Hiroshima University, Japan. Healthy and inflamed gingival tissue samples were collected from the Clinic of Periodontology at the Hiroshima University Hospital. Healthy gingival tissue, characterized by a lack of bleeding on probing (gingival pocket depth: 3 mm), was collected from a healthy woman volunteer aged 43 years who provided informed consent prior to enrollment. Inflamed gingival tissues were obtained from diseased sites, characterized by radiological bone resorption and bleeding on probing (gingival pocket depth, 7 mm; man, aged 73 years), of patients with periodontal disease who had undergone flap surgery.

### 2.3 Detection of LRG1 in serum, GCF, and gingival tissue

Enzyme‐linked immunosorbent assay (ELISA) was performed using an LRG1 Assay Kit (Cat#27769 for humans, Cat#27785 for mice, IBL, Gunma, Japan) to determine the serum, GCF, or gingival tissue LRG1 levels. Hundred‐fold preservative serum, eluted GCF, or homogenized mouse gingival tissue was added to the capture antibody‐immobilized plate and incubated at 4°C for 18 h for the human samples and at 37°C for 1 h for the mouse samples. Detection antibodies were added after washing thrice with PBST and incubated at 37°C for 30 min for the human samples and at 4°C for 30 min for the mouse samples. A substrate solution was added for detection, and the reaction was terminated after incubating at room temperature (RT) for 30 min. H_2_SO_4_ (2 N) was added subsequently for color development, which was measured using an ELISA reader (OD_405_, Bio‐Rad Laboratories). The target concentration was calibrated using a standard curve prepared via serial dilution. Each sample was examined in triplicate using a 96‐well ELISA plate. The limits of detection for the analytes were as follows: 1.56 ng/mL; human LRG1, 0.25 ng/mL; mouse LRG1.

Frozen sections of each tissue (healthy and inflamed tissues) were fixed with acetone‐alcohol at 4°C for 1 min for the detection of LRG1 in gingival tissue. Each section was incubated with 2 μg/mL of anti‐LRG1 rabbit monoclonal antibody (#ab178698, Abcam, Cambridge, UK) in phosphate‐buffered saline (PBS) at 4°C for 12 h after blocking with 0.5% BSA in PBST. Normal rabbit IgG was used as the negative control. Peroxidase conjugated with anti‐mouse IgG (Jackson ImmunoResearch, PA, USA) was applied at RT for 30 min. A DAB‐chromogen solution was added to initiate a colorimetric reaction and incubate it for 10 min. The nuclei were stained with hematoxylin (Sigma‐Aldrich). A microscope (BZ‐9000; Keyence, Japan) was used to detect the signals in the sections.

### 2.4 Cell culture (HGEC and HGF)

Human gingival epithelial cells (HGEC; #PCS‐200‐014) were purchased from American Type Culture Collection (ATCC) and sub‐cultured in Dermal Cell Basal Medium (PCS‐200‐030, ATCC) supplemented with Keratinocyte Growth Kit (PCS‐200‐040, ATCC) and 100 U penicillin/streptomycin (P4333‐100ML, Sigma‐Aldrich) in humidified air with 5% CO_2_ at 37°C. Subsequent experiments were performed using confluent cultures of HGECs. Human gingival fibroblasts (HGF) were obtained from healthy volunteers as described previously (Fujita et al., 2007) and cultured to confluence in complete Dulbecco's modified Eagle's medium (DMEM; Sigma‐Aldrich) supplemented with 10% fetal bovine serum (Invitrogen, Carlsbad, CA, USA), L‐glutamine (Sigma‐Aldrich), and antibiotics (penicillin, streptomycin, and gentamicin; Invitrogen). Cells were harvested with 0.05% trypsin and 0.02% ethylene diamine tetrameric acid (EDTA) after the HGF cells formed a confluent monolayer and transferred to a 100‐mm diameter plastic culture dish. The subsequent experiments were performed using fourth‐passage HGF cultures.

### 2.5 Preparation of bacteria and stimulation of cell

*P. gingivalis* 33277 (10^8^ CFU/mL), TGF‐β (10 ng/mL, Cat#210‐TA‐005, R&D systems, MN, USA), or TNF‐α (10 ng/mL, Cat#210‐TA‐005, R&D systems) was used to stimulate confluent cultures of HGEC or HGF, and the mRNA expression of LRG1 or IL‐6 was determined after 12 h. The bacterial strain was purchased from *ATCC* and prepared as described in a previous study (Ouhara et al., 2018).

### 2.6 Real‐time polymerase chain reaction

Extraction of the total RNA from the cells after stimulation was performed using RNAiso (Takara Bio Inc.) in accordance with the manufacturer's protocol. A two‐step real‐time polymerase chain reaction (RT‐PCR) system was used to analyze the extracted total RNA (Ouhara et al., 2012). Reverse transcription (RT) of 1 μg of total RNA was performed using ReverTra Ace® qPCR RT Master Mix (TOYOBO, Osaka, Japan). Real‐time PCR was performed using 1 μL of cDNA according to the amplification conditions described previously (Ouhara et al., 2012). StepOnePlus (Applied Biosystems) was used to perform real‐time PCR. Template cDNA (1 μL) was mixed with 4 μL of the Core Reagent Fast SYBR® Master Mix system (Applied Biosystems), 4.5 μL of distilled water, and 0.5 μL of primer. The following primer sets were used: LRG1 forward, 5′‐GCGCCTCTAAGCTCCAAGAA‐3′ and LRG1 reverse, 5′‐GTTCTCCCCAAGGTCAAGGG‐3′; IL‐6 forward, 5′‐GGAGACTTGCCTGGTGAAAA‐3′ and IL‐6 reverse, 5′‐GTCAGGGGTGGTTATTGCAT‐3′; β‐actin forward, 5′‐GACGGGGTCACCCACACTGT‐3′ and β‐actin reverse, 5′‐AGGAGCAATGATCTTGATCTTC‐3′.

### 2.7 Induction of periodontal disease in mice

All experimental procedures involving animals performed in this study were approved by the Ethics Committee of Hiroshima University (approval no. A22‐94‐2). As described in a previous report (Tamura et al., 2022), Pg 33277 was inoculated (10^8^ bacterial cells/50 μL in 2% carboxymethylcellulose (CMC) solution) twice a week for 6 weeks to C57BL/6JJcl mice (Pg group), and the state of the periodontal tissue was evaluated. The same volume of CMC was used as the negative control (control group). LRG1 was injected into the palatal gingiva between the first, second, and third molars using a 31G‐needle syringe on day 0 (1 mg/mL, 2 uL/site, LRG1 group) to evaluate the effect of LRG1 on the resorption of alveolar bone. The same volume of PBS was used as the negative control (PBS group). The periodontal tissue was evaluated after 2 weeks. An anti‐mouse IL‐6 receptor (IL‐6R) antibody (MR16‐1, 0.8 mg/kg) was injected intraperitoneally at the time of *P. gingivalis* oral inoculation (day 0) for the inhibition of IL‐6 signaling. Normal mouse IgG was used as the negative control. MR16‐1 was provided by Chugai Pharmaceutical (Tokyo, Japan; Akita et al., 2017). Serum samples were collected from the caudal vein of the mice. The periodontal tissue samples obtained from C57BL/6JJcl mice were lysed in 500 μL of PBST with 0.1% PMSF and 1% proteinase inhibitor cocktail and homogenized using a homogenizer. The root surface of the maxillary bone was stained with methylene blue, and the extent of bone resorption was determined as described in a previous study (Kawai et al., 2007).

The upper jaw isolated at the end of the experiment was incubated in 4% paraformaldehyde for 24 h, decalcified in 10% EDTA for 14 days, and embedded in paraffin. Tissue sections of 7 μm thickness were stained with hematoxylin and eosin or a TRAP staining kit (TRAP/ALP Stain Kit; FUJIFILM Wako Chemicals, Tokyo, Japan).

### 2.8 Detection of IL‐6 and anti‐*P. gingivalis* antibody in mice

ELISA for mouse IL‐6 (#431304; BioLegend Inc., San Diego, CA, USA) was used to detect IL‐6 in the serum and homogenized tissue in accordance with the manufacturer's instructions. A solid‐phase anti‐IL‐6 monoclonal antibody (diluted in coating buffer to a final concentration of 1 μg/mL) was coated onto a 96‐well ELISA plate (BD Falcon, Franklin Lakes, NJ, USA) to capture the target. The supernatant or standard (diluted in PBST from 1 ng/mL to zero) was added to each well after blocking each with 1% BSA in PBST. HRP conjugated with anti‐IgG (2000‐fold dilution in PBST) was applied to the wells after the application of the detection antibody (diluted in PBST to a final concentration of 1 μg/mL). The limits of detection for the analytes were as follows: 15.6 pg/mL for mouse IL‐6. Antibody titers against *P. gingivalis* were measured by ELISA. Whole *P. gingivalis* cells in sodium bicarbonate buffer (pH 9.4) were coated onto 96‐well Maxisorp Nunc Immunoplates (Nunc, Roskilde, Denmark) and incubated overnight at RT. Mouse serum (100‐fold dilution) was added to each well at RT for 2 h after blocking each well with 1% BSA in PBS supplemented with 0.05% Tween 20 (PBST) at RT for 1 h. The wells were washed thrice with PBST and incubated with a goat horseradish peroxidase‐conjugated secondary antibody (2000‐fold dilution in PBST) at RT for 1 h. Citrate–phosphate buffer (pH 5.0) containing 0.3% hydrogen peroxide and 0.25% o‐phenylenediamine was added after the final wash. The coloring reaction was continued for 15 min and terminated by adding 25 μL of 2 N sulfuric acid. The absorbance was measured at 405 nm using an ELISA plate reader (Bio‐Rad Laboratories).

A standard curve prepared from serial dilutions was used to calibrate the target calibration. Each sample was examined in triplicate using a 96‐well ELISA plate.

### 2.9 Statistical analysis

All experiments were performed independently in triplicate. The data are presented as the mean ± SD. The normality of the data distribution in each dataset was assessed using the Shapiro–Wilk test. Non‐normally distributed data were analyzed using a two‐tailed unpaired Student's *t*‐test or Mann–Whitney *U* test. Multiple comparisons were performed using the Bonferroni‐corrected Mann–Whitney *U* test. The correlation between the serum LRG1 levels and PISA was analyzed using Spearman's rank correlation coefficient. A *p*‐value of <0.05 was considered statistically significant in all analyses.

## 3 RESULTS

### 3.1 Correlation between PISA and the serum LRG1 levels of the patients with periodontal disease

**FIGURE 1:**
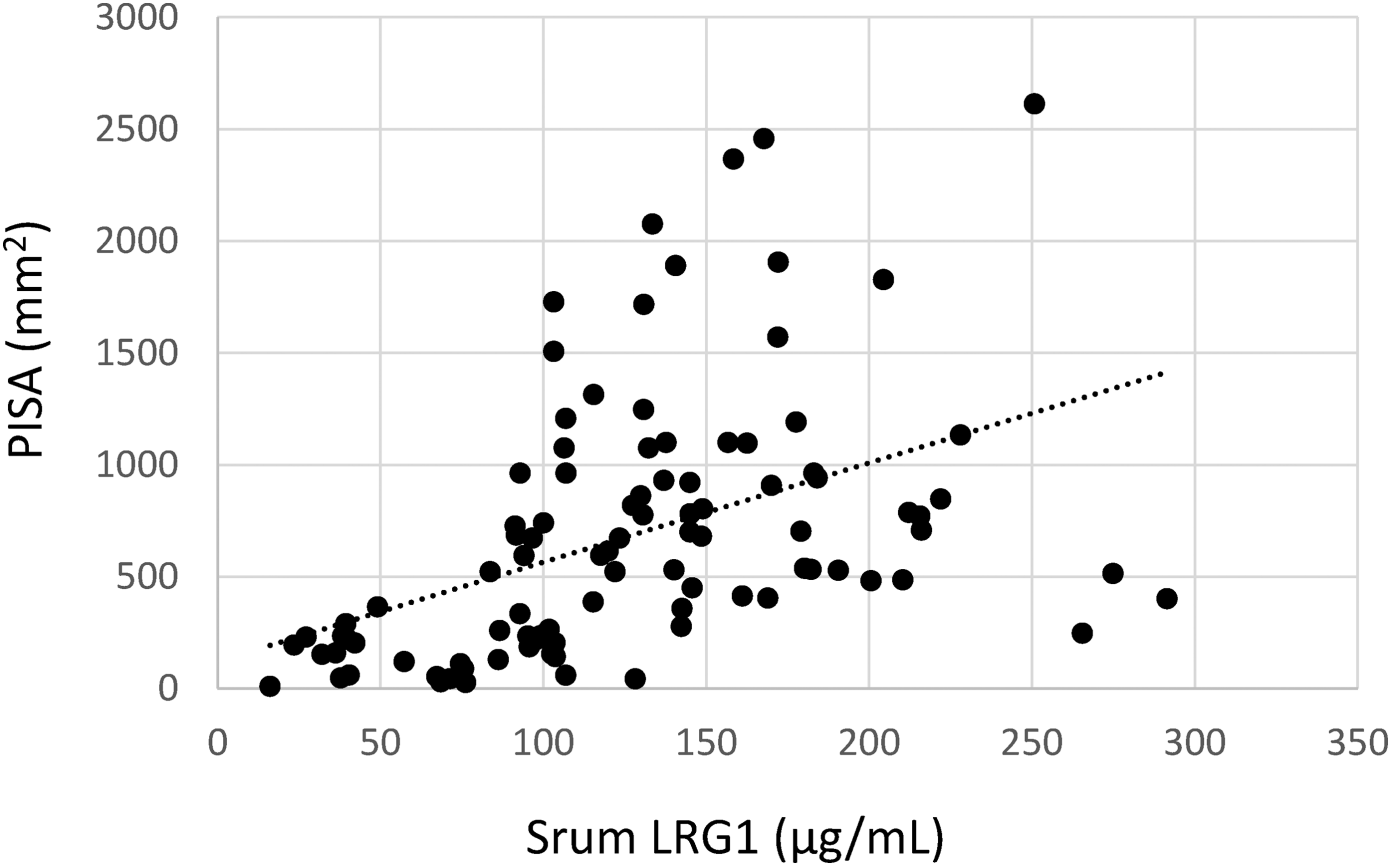
Correlation of periodontal inflamed surface area (PISA) and leucine‐rich α‐2‐glycoprotein‐1 (LRG1) in serum. CAL and bleeding on probing (BOP) of periodontitis patients were measured at the first visit to determine the correlation between PISA and LRG1 in the serum. Blood was collected from the participants' periphery with consent. The PISA was calculated as described in previous studies. The serum LRG1 levels were measured using ELISA. The *x*‐axis represents the serum LRG1 levels, and the vertical axis represents the PISA. The PISA and LRG1 levels were analyzed using Spearman's rank correlation coefficients.

Inflammation of the periodontal tissue is correlated with the inflammatory cytokine levels, such as IL‐6, in the serum (Tonguç et al., 2022). The correlation between PISA, which quantifies the inflammatory area of periodontal pockets, and the LRG1 levels in the serum was evaluated to investigate the relationship between periodontitis and LRG1. A positive correlation was observed between PISA and LRG1 expression (Spearman's rank correlation coefficient, rs = 0.60; Figure 1). LRG1 in GCF was measured in two patients with high PISA and two with low PISA, chosen as representatives regarding PD of 4 mm or more and BOP‐positive sites. In addition to the correlation between serum LRG1 and PISA, the high PISA group had higher LRG1 abundance in GCF than the low PISA group (Table 1).

**TABLE 1:** Leucine‐rich α‐2‐glycoprotein‐1 (LRG1) levels in CGF.

Pt	LRG1 in GCF (ng/μL)	LRG1 in serum (ng/mL)	PD (mm)	BOP	PISA (mm^2^)
1	15.52	39.90	6	+	207.8
2	10.24	36.23	4	+	176.35
3	29.01	222.00	9	+	824.73
4	21.99	177.60	7	+	1200.12

### 3.2 Immunohistological observation of LRG1 in human gingival tissue

**FIGURE 2:**
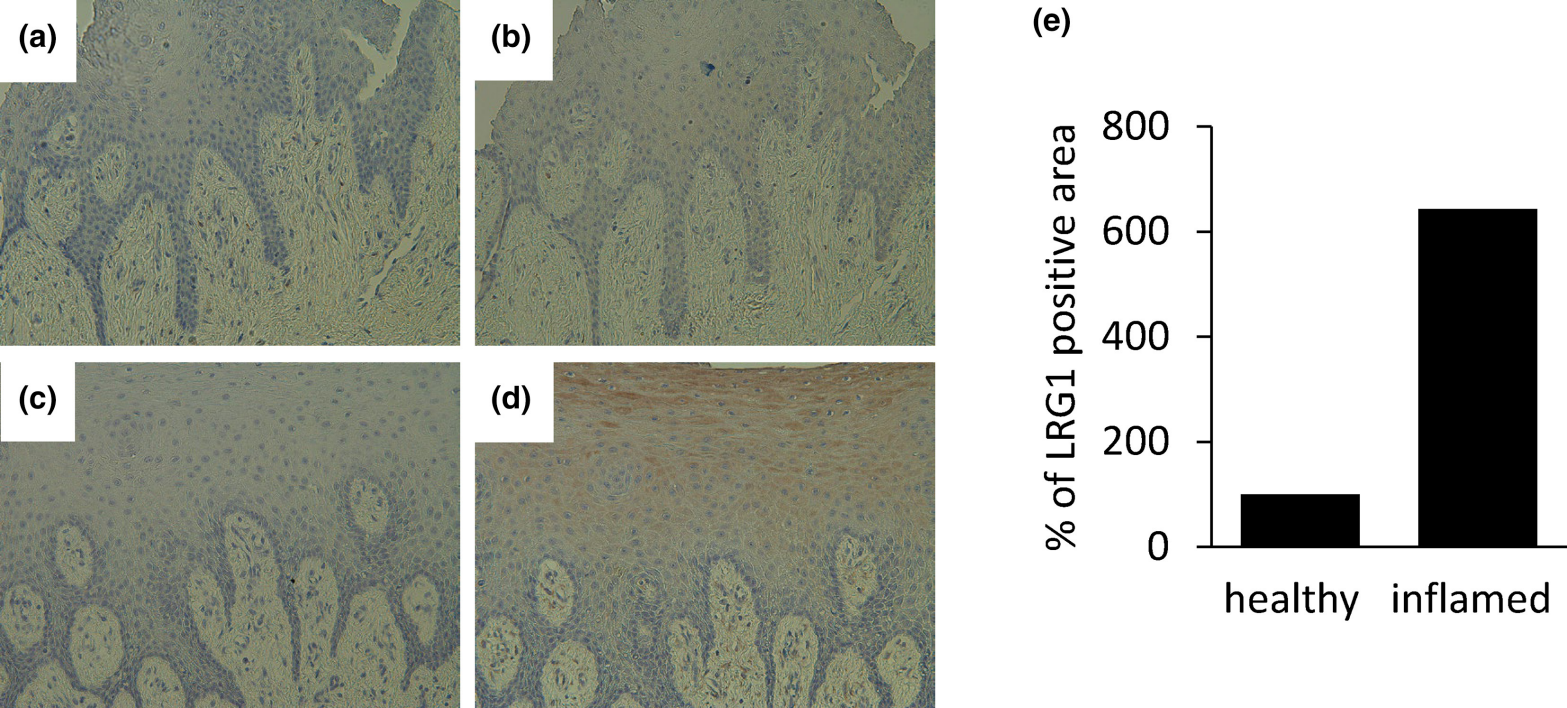
Leucine‐rich α‐2‐glycoprotein‐1 (LRG1) localization in periodontal tissue. The localization of LRG1 in the periodontal tissues of healthy volunteers and patients with periodontitis was investigated via immunohistochemical staining. (a, b) Healthy volunteers. (c, d) Patients with periodontitis. (a, c) Images depicting staining with a control antibody. (b, d) Images depicting staining with an anti‐LRG1 antibody. Original magnification ×200; scale bar: 100 μm. (e) Quantification of color development of immunohistochemical staining. The graph presents the ratio of patients with periodontitis to healthy volunteers.

Localization of LRG1 in human gingival tissues obtained from healthy volunteers and patients with periodontitis was determined. An area in the epithelial layer and connective tissue of the inflamed tissue obtained from patients with periodontitis stained strongly positive for LRG1 antibody (Figure 2d). LRG1 was expressed around the capillary vessels of the connective tissue. Weak LRG1 staining was observed in the epithelial layer and connective tissues of the healthy gingival tissue (Figure 2b). Normal IgG was not observed in any of the areas that stained positive in either tissue (Figure 2a,c). Immunostaining‐positive areas were quantified using ImageJ software. The area of the inflamed tissue that stained positive was 6.42 times more positive areas than that of the healthy tissue that stained positive (Figure 2e).

### 3.3 mRNA expression of LRG1 and IL‐6 in cultured cell

**FIGURE 3:**
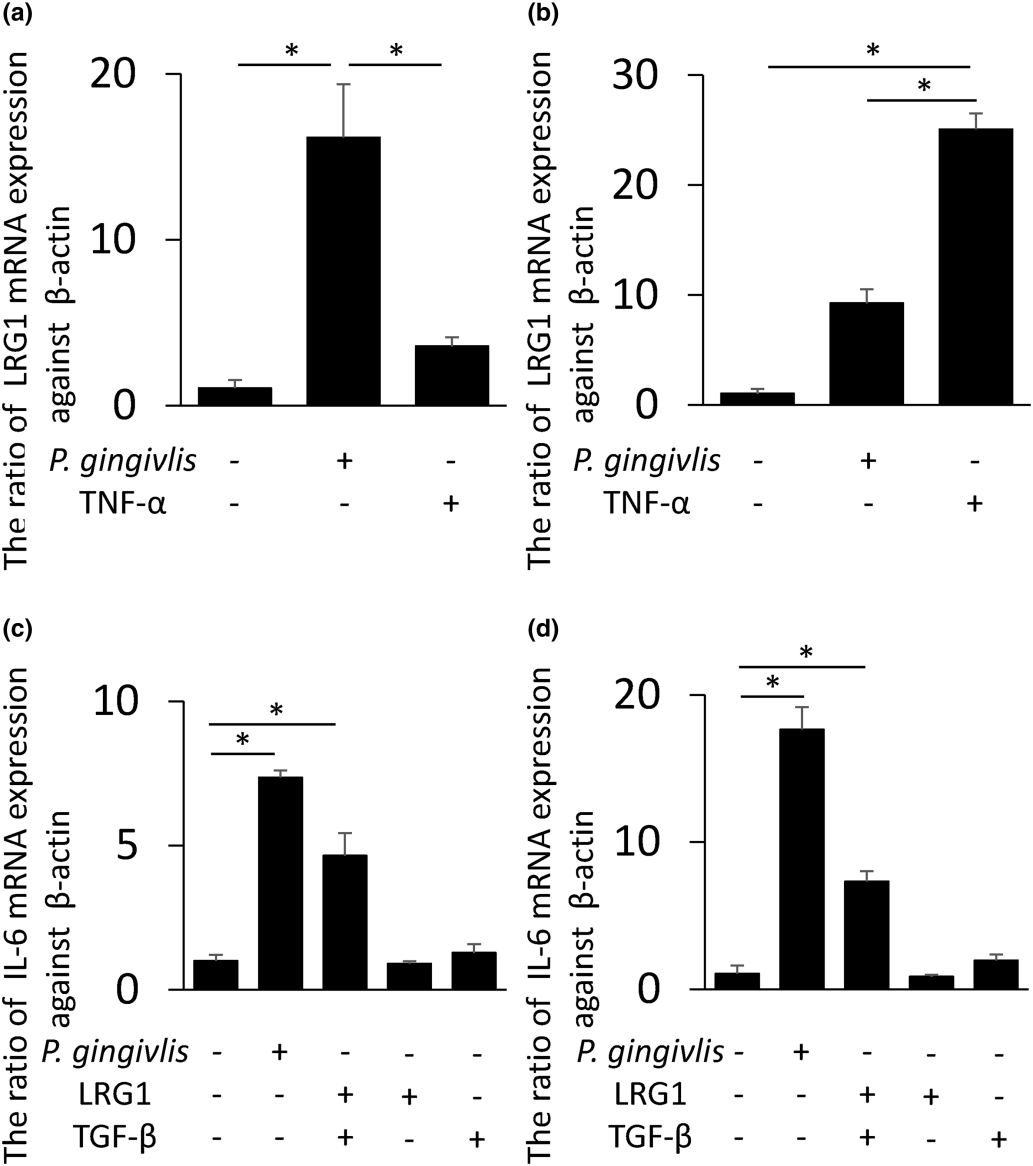
mRNA expression of leucine‐rich α‐2‐glycoprotein‐1 (LRG1) and IL‐6 in human gingival epithelial cells (HGEC) and human gingival fibroblasts (HGF). HGEC (a) and HGF (b) are stimulated with *Porphyromonas gingivalis* (10^7^ CFU/mL) or TNF‐α (10 ng/mL) for 12 h to determine mRNA expression of LRG1. IL‐6 mRNA expression when HGEC (c) and HGF (d) are co‐stimulated with LRG1 and TGF‐β for 12 h to determine mRNA expression of IL‐6. The ratio of stimulation to no stimulation is shown. *Significantly higher mRNA expression of LRG1 and IL‐6 in the student's *t*‐test (*p < 0.05).

mRNA expressions in the HGEC and HGF were determined in the presence and absence of *P. gingivalis* or TNF‐α to identify the source of LRG1. LRG1 mRNA expression was induced by Pg and TNF‐α in HGEC and HGF (*P. gingivalis* stimulation: 16.2‐, 9.3‐fold increase, TNF‐α stimulation: 3.6‐, 25.1‐fold increase, respectively, Figure 3a,b). Co‐stimulation of LRG1 and TGF‐β is known to induce the production of inflammatory cytokines (Wang et al., 2013). Consequently, IL‐6 mRNA expression in HGEC and HGF was evaluated in the presence of LRG1 and TGF‐β. IL‐6 expression in HGEC or HGF was not induced via single stimulation with LRG1 or TGF‐β. However, the combination of LRG1 and TGF‐β induced IL‐6 in the same intensity as bacterial stimulation (4.7‐ and 7.3‐fold increase, respectively, Figure 3c,d).

### 3.4 Assessment of periodontal disease in mice

**FIGURE 4:**
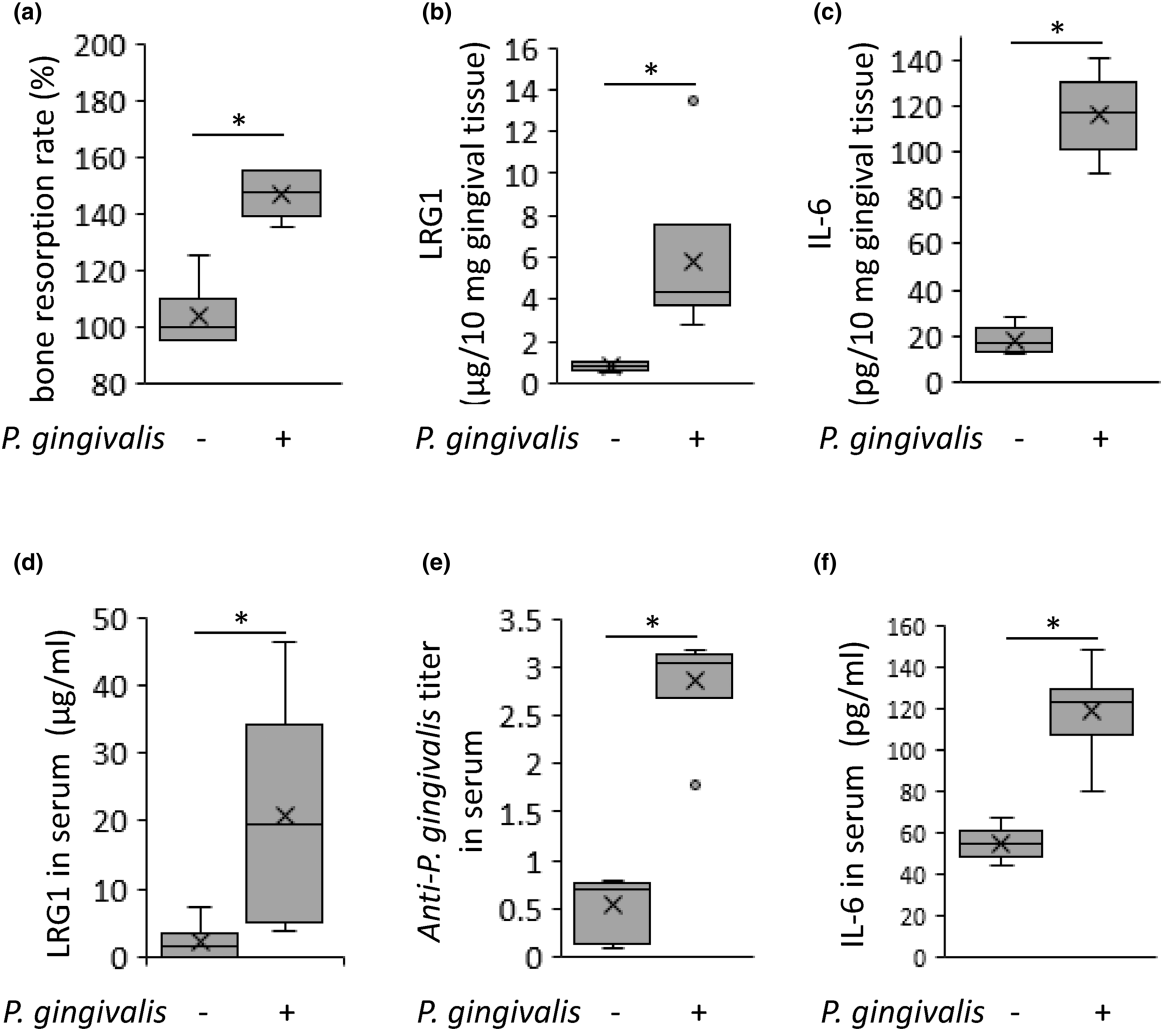
Evaluation of *Porphyromonas gingivalis* orally inoculated mouse periodontitis model. *P. gingivalis* suspended in carboxymethylcellulose (CMC) was applied to the oral cavity of C57BL6j mice twice a week for 6 weeks. Alveolar bone resorption (a), leucine‐rich alpha‐2 glycoprotein (LRG) in the periodontal tissues (b), IL‐6 in the periodontal tissues (c), LRG1 in the serum (d), anti‐*P. gingivalis* antibody in the serum (e), and evaluated IL‐6 in the serum (f) in the *P. gingivalis* application group compared with those in the CMC application group. *p < 0.01; Mann–Whitney *U* test.

C57/BL6j mice were divided into two groups (control and Pg). Evaluation of maxillary bone resorption 6 weeks after Pg infection revealed that greater bone resorption was induced in the Pg‐infected group compared with that in the control group (45.7% increase, Figure 4a). Evaluation of the LRG1 level in the palatal gingiva and serum evaluated using ELISA revealed that the values in the Pg‐infected group were higher than those in the control group (gingival tissue: 237.8% increase, serum: 753.0% increase, respectively, Figure 4b,d). The IgG titer against *P. gingivalis* whole cells in serum was higher in the Pg‐infected group (390.9% increase; Figure 4e). The IL‐6 levels in the gingival tissue and serum of the Pg‐infected group were higher than those in the Ctrl group (gingival tissue: 603.6% increase, serum: 114.4% increase, respectively, Figure 4c,f).

### 3.5 Direct effect of LRG1 in periodontal tissue

**FIGURE 5:**
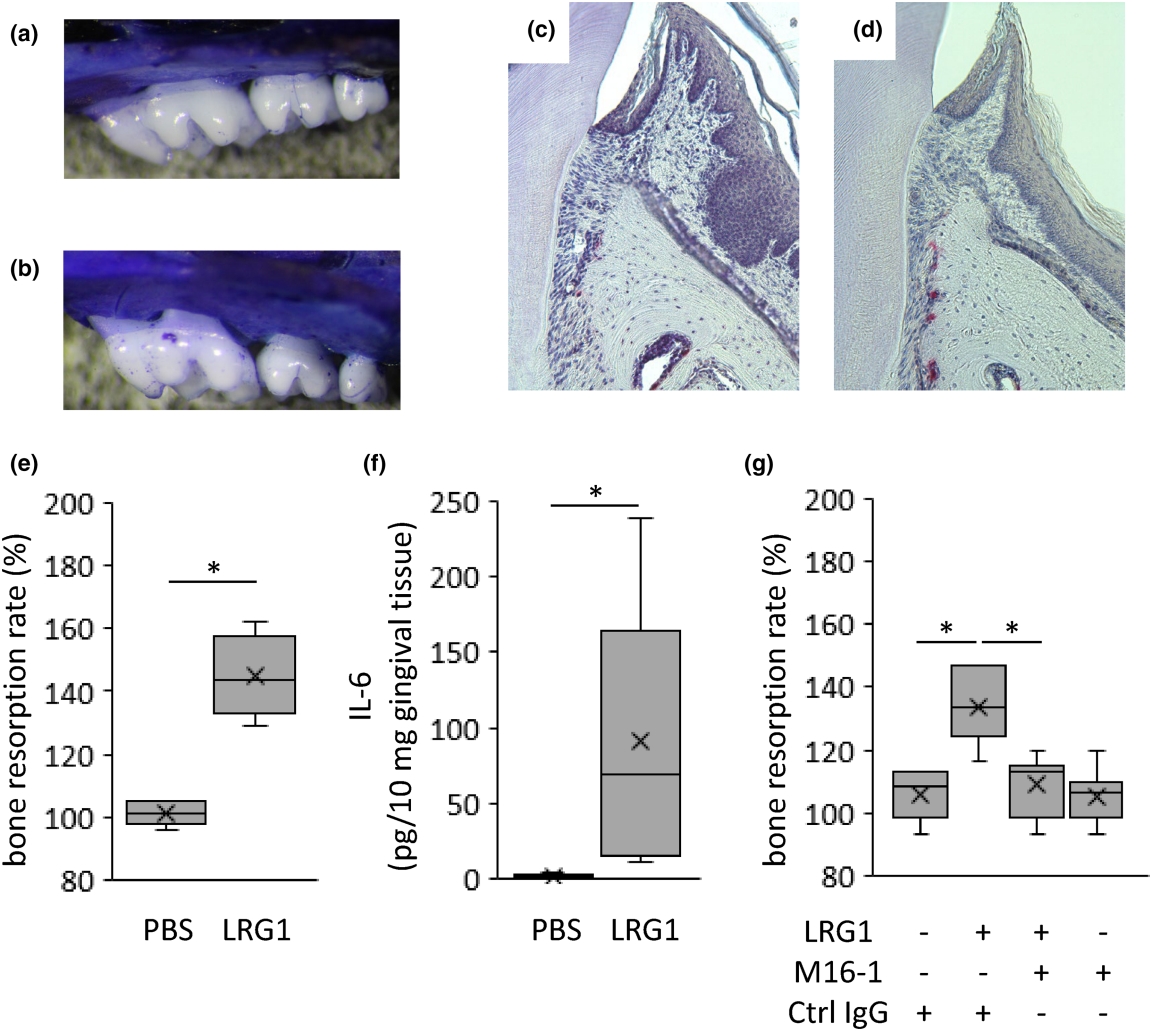
Leucine‐rich α‐2‐glycoprotein‐1 (LRG1) induces bone resorption in mouse gingival tissue. (a) Phosphate‐buffered saline (PBS) or (b) LRG1 injected between the first and second molars and between the second and third molars of mice (LRG1 1 mg/mL, 2 uL/site). Evaluation of alveolar bone resorption in mice. Histological evaluation of mouse maxillary periodontal tissue (c PBS injection, d LRG1 injection). Alveolar bone resorption (e); IL‐6 in periodontal tissues (f) of the mice with the palatal gingival injection of LRG1 or PBS were determined. (g) The amount of alveolar bone resorption in the mice that received intraperitoneal administration of anti‐mouse IL‐6R antibody (MR16‐1) and IgG in the mice that received palatal gingival injection of LRG1 or PBS. Original magnification ×100; scale bar, 100 μm. *p < 0.01; Mann–Whitney *U* test.

Recombinant LRG1 was injected into the palatal tissue to determine the direct effect of LRG1. LRG1 induced periodontal bone loss (43.8% increase; Figure 5a,b,e). The periodontal tissue samples of the mice in the control and LRG1‐injected groups were stained with TRAP. TRAP‐positive cells were predominantly observed along bone resorption sites in the LRG1‐injected group (Figure 5c,d). LRG1 also exacerbated the production of IL‐6 in the gingival tissue (29.6‐fold increase; Figure 5f) and was involved in IL‐6 induction (Figure 3c,d). Therefore, whether LRG1‐induced alveolar bone resorption in mice was due to the direct action of LRG1 or the indirect action of IL‐6 using an anti‐mouse IL‐6R antibody (MR16‐1) was also examined in this study. The systemic administration of an IL‐6 receptor monoclonal antibody was found to inhibit LRG1‐induced alveolar bone resorption (Figure 5g). No change in bone resorption was observed after antibody administration alone compared with the control group.

## 4 DISCUSSION

The findings of the present study demonstrated that serum LRG1 levels are correlated with PISA, the inflammatory surface area in patients with periodontitis. LRG1 production in the periodontal tissues was also found to be higher than that in healthy tissues. Furthermore, the amount of LRG1 in GCF in BOP‐positive pockets, indicating active pockets, was higher in patients with high PISA values than in patients with low PISA values. In vitro experiments revealed that *P. gingivalis* and TNF‐α stimulated LRG1 expression in HGEC and HGF. Similarly, *P. gingivalis* infection increased the amount of LRG1 in periodontal tissue and serum in the mouse periodontitis model.

Previous studies have shown that basic periodontal treatment effectively reduces clinical symptoms and LRG1 levels in the serum and GCF of patients with grade III moderate periodontitis (Keles Yucel & Balli, 2021). This study establishes a correlation between serum LRG1 levels and periodontal disease based on PISA, suggesting its potential as an effective serum marker for evaluating periodontal disease (Figure 1). PISA is calculated using the sum of the area of the BOP‐positive region among periodontal pocket areas (Nesse et al., 2008). LRG1 influences epithelial repair in patients with ulcerative colitis (Camilli et al., 2022). LRG1 also inhibits IL‐22‐mediated tissue destruction at the sites of inflammation (Camilli et al., 2022). Thus, overproduction of LRG1 may exacerbate periodontal pocket inflammation at BOP‐positive active sites and inhibit wound healing. Future studies must aim to investigate the mechanism through which the wound healing of periodontal tissues caused by LRG1 is delayed.

Bacterial components and inflammatory cytokines induce periodontal inflammation (Hajishengallis, 2015). LRG1 induction involves the recognition of bacterial components by host recognition protein receptors, such as toll‐like receptors and nucleotide‐binding oligomerization domain, leading to the production of pro‐inflammatory cytokines, such as IL‐6 (Dritsoula et al., 2022; Fujimoto et al., 2020). Excessive inflammatory response in periodontal disease is characterized by bone destruction, similar to RA, the pathophysiology of which also involves LRG1 production (Fujimoto et al., 2015). Therefore, the present study hypothesized that LRG1 may be involved in alveolar bone resorption. The injection of LRG1 into mouse gingival tissue resulted in bone resorption and an increase in IL‐6 in the gingival tissue as a direct effect (Figure 5a–f). Osteoclast formation is caused by signals mediated by NFATc1 (Takayanagi, 2021). In vitro data suggest that co‐stimulation by LRG1 and TGF‐β induces the production of IL‐6 (Figure 3c,d). Osteoclast activation is likely mediated by IL‐6 (Yokota, 2023). Therefore, the present study focused on osteoclast activation by the ancillary pathway of RANKL via IL‐6 and examined whether bone resorption induced by LRG1 was suppressed in vivo by an IL‐6R neutralizing antibody (Figure 5g). The findings suggest that elevation of LRG1 by bacteria, induction of IL‐6, and activation of the Receptor activator of nuclear factor‐kappa B ligand (RANKL) pathway by the inflammatory cascade promote osteoclast differentiation and bone resorption. However, other studies have reported that IL‐6 induces the production of RANKL (Hashizume et al., 2008). Thus, it is necessary to investigate whether the IL‐6‐IL‐6R signal affects osteoclast differentiation in a direct manner or participates indirectly by increasing RANKL production.

Systemic and local elevation of LRG1 levels has been observed in patients with systemic diseases, such as diabetes mellitus and RA, suggesting a correlation with the pathology of the disease and demonstrating its effectiveness as a new diagnostic marker (Camilli et al., 2022; Fujimoto et al., 2015; Hong et al., 2019). LRG1 is synergistically affected in the progression of diabetic kidney disease (DKD) via TGF‐β/activin receptor‐like kinase 1 (ALK1) signaling (Hong et al., 2019). LRG1 also functions as a pro‐inflammatory factor and promotes the progression of RA. LRG1 promotes the activation of Th17 cells and Th17‐related cytokines, TNF‐α, and IL‐6. Moreover, it also induces severe joint destruction (Kim et al., 2013; Sarkar et al., 2010). Increased levels of IL‐6 at the diseased site induced by LRG1 sustain the formation of pro‐inflammatory Th17 cells (McGeachy et al., 2007). The upregulation of LRG1 in periodontal tissue may influence the progression of these systemic diseases via hematogenous migration. The promotion of wound healing and its influence on the proliferation of epithelial cells, fibroblasts, and vascular endothelial cells via the TGF‐Smad1/5/8 pathway are the physiological functions of LRG1 (Hong et al., 2019). However, appropriate ECM is not produced as inflammation persists, excessive LRG1 production occurs, and epithelial tissue and capillaries are not formed (Camilli et al., 2022). LRG1 produced locally in the periodontal tissue is translocated throughout the body, leading to an increase in serum LRG1 levels. The systemic transfer of bacterial components and cytokines into the blood circulation activates the inflammatory immune response (Konkel et al., 2019), and it is also possible that the transfer of inflammatory substances influences the upregulation of LRG1 throughout the body. Analysis of limited GCF samples indicated that patients with high serum LRG1 levels and PISA values also tended to have high LRG1 levels in GCF. However, these data do not confirm whether LRG1 produced locally in periodontal tissue is distributed throughout the body. Therefore, it remains unclear whether serum LRG1 is effective for evaluating periodontal disease. This limitation underscores the need for future studies to investigate the amount of LRG1 in GCF as a biomarker in the pathology of periodontitis and for evaluating the treatment stage of periodontitis. The findings of the present study suggest that LRG1 is derived from epithelial cells and gingival fibroblasts; however, there are reports that it is derived from lymphoid cells (De Rossi et al., 2022). Thus, it is necessary to investigate the major source of LRG1 that plays a dominant role in the progression of periodontitis. Although the involvement of LRG1 has been reported, it is necessary to investigate the interaction between periodontitis and the pathology of periodontal disease‐related diabetes and RA.

## AUTHOR CONTRIBUTIONS

**Kazuhisa Ouhara:** Writing – original draft; writing – review and editing; funding acquisition; data curation; conceptualization. **Tasuku Takemura:** Data curation. **Yuri Taniguchi:** Data curation; methodology. **Ryousuke Fujimori:** Data curation; visualization. **Tetsuya Tamura:** Data curation; investigation. **Yuki Akane:** Data curation. **Shinji Matsuda:** Formal analysis. **Yuta Hamamoto:** Data curation. **Tomoaki Shintani:** Data curation. **Mikihito Kajiya:** Supervision; methodology; validation. **Syuichi Munenaga:** Data curation. **Tomoyuki Iwata:** Visualization; data curation. **Tsuyoshi Fujita:** Supervision. **Noriyoshi Mizuno:** Writing – review and editing; supervision.

## FUNDING INFORMATION

This research was supported by Grants‐in‐Aid for the Encouragement of Young Scientists (20K18537, 21K16970, and 22K17038) and Scientific Research (C) (22K09983) from the Japan Society for the Promotion of Science.

## CONFLICT OF INTEREST STATEMENT

The authors declare no conflict of interest associated with this manuscript.

## CLINICAL TRIAL REGISTRATION

The authors declare no clinical trial registration associated with this manuscript.

## Data Availability

All data generated or analyzed during this study are included in the published article.
